# The correlation between the expression of genes involved in drug metabolism and the blood level of tacrolimus in liver transplant receipts

**DOI:** 10.1038/s41598-017-02698-w

**Published:** 2017-06-13

**Authors:** Jianhai Wang, Keqiu Li, Xiaoning Zhang, Dahong Teng, Mingyan Ju, Yaqing Jing, Yuxia Zhao, Guang Li

**Affiliations:** 1 Basic Medical College, Tianjin Medical University, Tianjin, 300070 China; 2 Department of Hepatobiliary and Liver transplantation Surgery, Tianjin First Central Hospital, Tianjin, 300192 China

## Abstract

Immunosuppressive medications, such as tacrolimus and mycophenolate mofetil, are commonly used for reducing the risk of organ rejection in receipts of allogeneic organ transplant. The optimal dosages of these drugs are required for preventing rejection and avoiding toxicity to receipts. This study aimed to identify the correlation between the expression profiling of genes involved in drug metabolism and the blood level of tacrolimus in liver transplant receipts. Sixty-four liver transplant receipts were enrolled in this retrospective study. Receipts were divided into low (2–5.9 ng/ml) and high (6–15 ng/ml) tacrolimus groups. Clinical assessment showed that the blood level of tacrolimus was inversely correlated with the liver function evaluated by blood levels of total bilirubin and creatinine. Compared to the high tacrolimus group, expression levels of six cytochrome P450 enzymes, CYP1A1, CYP2B6, CYP3A5, CYP4A11, CYP19A1, and CYP17A1 were significantly higher in the low tacrolimus group. The expression levels of these genes were negatively correlated with the tacrolimus blood level. Enzyme assays showed that CYP3A5 and CYP17A1 exerted direct metabolic effects on tacrolimus and mycophenolate mofetil, respectively. These results support clinical application of this expression profiling of genes in drug metabolism for selection of immunosuppressive medications and optimal dosages for organ transplant receipts.

## Introduction

Liver transplantation is a common therapy for patients with chronic and acute liver diseases that lead to life-threatening complications^1^. Graft-versus-host disease is a serious complication that can occur after solid organ transplantation^2^. Immunosuppressive therapy, including tacrolimus, mycophenolate mofetil, and methylprednisolone has been widely used for solid organ transplantation to decrease the risk of organ rejection by suppressing transplanted donor cell-induced immune responses^3^. Tacrolimus is a calcineurin inhibitor, which has high interpersonal differences in pharmacokinetics and pharmacodynamic responses and is subject to drug-drug and drug-disease interactions^4, 5^. It has been reported that tacrolimus treatment has narrow therapeutic index window^6^. Underexposure of tacrolimus in the acute posttransplant period increases the risk of rejection, and overexposure causes considerable toxicity in receipts^4, 5^. Mycophenolate mofetil, a prodrug of mycophenolic acid, is an inhibitor of inosine-50-monophosphate dehydrogenase, an enzyme used in the de novo synthesis of guanosine nucleotides for lymphocyte activation^7^. Mycophenolate mofetil has higher oral bioavailability as compared to mycophenolic acid, however, the risk of side effects caused by this drug is the same as other immunosuppressive drugs^8^. Therefore, maintaining the optimal blood level of immunosuppressive drugs in a safe therapeutic window is critical for management of organ transplantation.

It is known that genetic factors, such as genes encoding drug metabolizing enzymes, play a critical role in regulation of the pharmacokinetic properties of immunosuppressive drugs, although several other factors, such as age, sex, body weight, and drug interactions may lead to the high interpersonal variability of drug metabolism^3^. Drug metabolizing enzymes include Phase I and Phase II enzymes. The human cytochrome P450 (CYP), a phase I enzyme family, is responsible for the metabolism of several endogenous compounds, drugs and other xenobiotic^9^. It has been reported that the major enzymes contributing to bioavailability and elimination of tacrolimus are isoforms of CYP3A, specifically CYP3A4 and CYP3A5^6^. Metabolism of mycophenolate mofetil from its active form to phenolic glucuronide, a pharmacologically inactive form, is mediated by glucuronidation of mycophenolic acid by a phase II enzyme, UDP glucuronosyl transferases^8^.

The majority of enzymes involved in drug metabolism are produced in the liver. During rejection in liver transplantation receipts, liver tissues are injured by the immune responses to the transplanted tissues and hepatic cells are released into the bloodstream^10, 11^. Thus the levels of drug metabolism enzymes in the blood may be associated with the graft function and the metabolic capacity of the liver. Studies have demonstrated that the P-450 mRNA expression level in peripheral blood is related to enzyme activity of P-450^12^ and the CYP3A4 gene expression level represents the metabolic capacity for tacrolimus^13^.

Therefore, this study aimed to identify the expression profiling of genes encoding drug-metabolizing enzymes in peripheral blood that is correlated with the blood level of tacrolimus in liver transplant receipts. We found that the expression levels of six cytochrome P450 enzymes, CYP1A1, CYP2B6, CYP3A5, CYP4A1, CYP17A1, and CYP19A1, were associated with the blood level of tacrolimus. This finding provides information for clinical application of the expression profiling of these drug metabolism-related genes to guide the appropriate selection of immunosuppressive medications and optimal dosages for organ transplant receipts.

## Results

### The blood level of tacrolimus is associated with the liver function in liver transplant receipts

This retrospective study included sixty-four liver transplant receipts who received a triple-drug immunosuppressive regimen, including tacrolimus, mycophenolate mofetil and methylprednisolone. To evaluate the relationship between the blood level of tacrolimus and the liver function in our study population, peripheral blood samples were collected before the morning dose at the third week after transplantation for examining the blood level of tacrolimus and clinical parameters for defining the liver function status. Generally, all receipts in this study had abnormal levels of glutamic-pyruvic transaminase (ALT), glutamic oxalacetic transaminase (AST), alkaline phosphatase (ALP), glutamyltransferase (GGT), total bilirubin and serum creatinine, which indicate pathological damage of the liver (Table 1).

**Table 1:** Demographic characteristics of recipients at the third week after liver transplantation.

Gender (female/male)	(15/49)
Blood type (A/B/AB/O)	(18/20/8/18)
Age (year)	51.29 ± 8.89
Bodyweight (kg)	71.87 ± 18.25
BMI (kg/m^2^)	24.64 ± 4.17
Tacrolimus dosage (mg/day)	2.56 ± 0.85 (0.5–4)
Albumin (g/dL)	37.63 ± 4.41
Hemoglobin (g/dL)	99.07 ± 21.90
ALT (U/L)	61.50 ± 64.77
AST (U/L)	30.47 ± 23.27
ALP (U/L)	144.51 ± 80.90
GGT (U/L)	88.41 ± 59.27
Total bilirubin (μmol/L)	29.08 ± 37.78
Creatinine (μmol/L)	79.00 ± 31.00
Urea nitrogen (mmol/L)	7.86 ± 5.56

Clinical parameters in blood are shown as Mean ± S.D.

**Figure 1:**
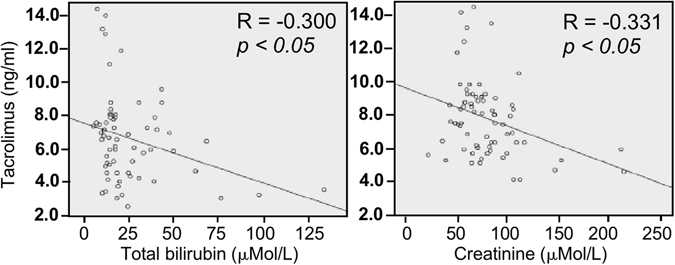
The correlation between the blood tacrolimus level and the blood levels of total bilirubin and creatinine in liver transplant receipts.

Our previous studies have demonstrated that hemoglobin, total bilirubin, and blood urea nitrogen after transplantation are significantly associated with the bioavailability of tacrolimus^14^. Clinical assessment of these liver transplant receipts revealed that the blood tacrolimus level was inversely related to the liver function evaluated by the blood levels of total bilirubin and creatinine (Fig. 1). These data suggest that the high blood level of tacrolimus may contribute to enhancing the ability of repressing the immune responses to graft rejection, leading to protection of the liver from damage. Thus, metabolism of tacrolimus and other immunosuppression medications play roles in maintaining the function of transplanted organs in receipts.

### Identification of an expression profiling of genes encoding drug-metabolizing enzymes in peripheral blood that is correlated with the blood level of tacrolimus in liver transplant receipts

It is well known that several enzymes mediate regulation of the metabolism of immunosuppression medications and drug-drug-interactions. We next examined whether there is a gene expression profiling involved in drug metabolism that is correlated to the blood levels of tacrulimus in organ transplant receipts. In this study, the range of the blood levels of tacrulimus in receipts was 2–15 ng/ml. These receipts were divided into low (2–5.9 ng/ml) and high (6–15 ng/ml) tacrolimus groups.

**Figure 2:**
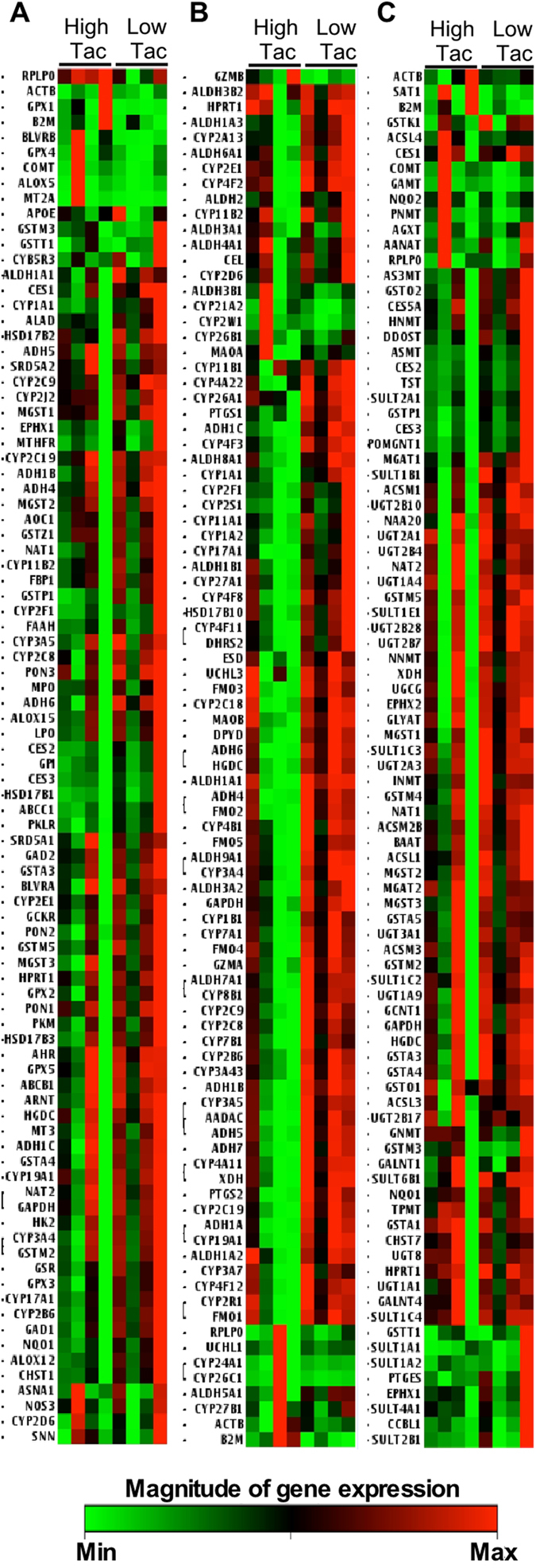
Heat map of three human drug metabolism PCR arrays. (**A**) Drug metabolism PCR array, (**B**) Phase I enzymes PCR array, (**C**) Phase II enzymes PCR array. Gene expression levels in low and the high tacrolimus (Tac) groups in liver transplant receipts are shown.

Human Drug metabolism RT^2^ Profiler PCR Array was performed using RNA isolated from peripheral blood samples of receipts. This array containing 84 genes encoding Phase I Enzymes and Phase II Enzymes involved in drug metabolism was applied for examining gene expression levels in four receipts from the low and four receipts from the high tacrolimus group (Fig. 2). Gene expression analysis showed that there were 26 genes with expression levels significantly increased for higher than 3 folds in the low tacrolimus group, as compared to those in the high tacrolimus group (p < 0.05) (Supplementary Table 1). These differently expressed genes encode 24 phase I enzymes, including cytochrome P450, aldehyde Dehydrogenase, alcohol dehydrogenase, esterase, prostaglandin-endoperoxide synthase, xanthine dehydrogenase, decarboxylases, glutathione transferases, N-acetyltransferases, sulfotransferases, and 2 phase II enzymes, SULT1B1 and GAD1.

**Figure 3:**
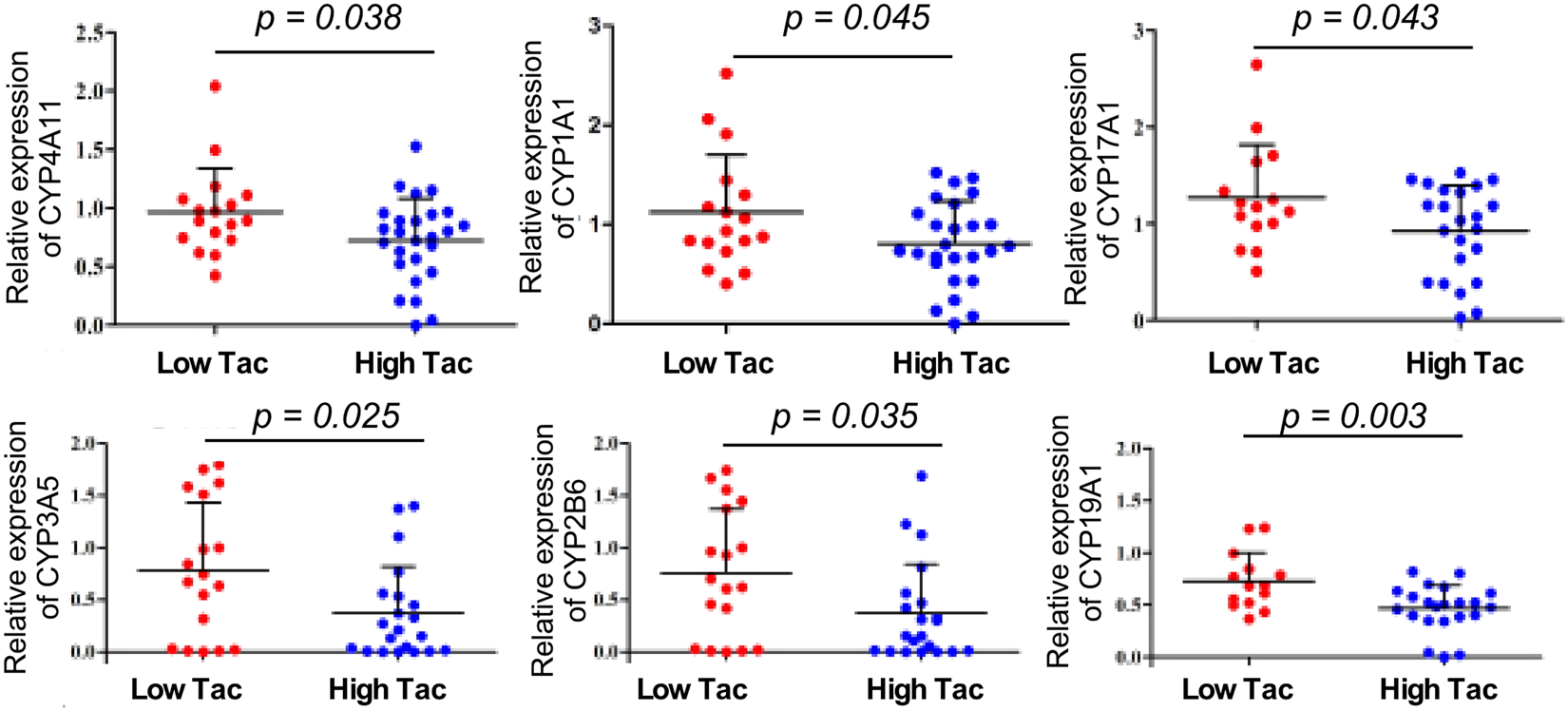
Validation of gene expression levels in the blood of low and the high tacrolimus (Tac) groups in liver transplant receipts. RNA from the blood of receipts was prepared for real-time PCR analysis of gene expression levels. Relative expression levels of indicated genes are shown. The average of the gene expression level from the low Tac group was set as 1, and gene expression levels in receipts were compared to this value.

To verify the results from RT^2^ Profiler™ PCR Array, we used custom-made RT² Profiler™ PCR Array for further analysis of expression levels of these genes in 64 liver transplant receipts. We found that 6 genes, CYP1A1, CYP2B6, CYP3A5, CYP4A11, CYP19A1, and CYP17A1 were significantly increased in the low tacrolimus group, as compared to those in the high tacrolimus group (p < 0.05) (Fig. 3). However, no significant difference of expression levels of the other 20 genes between low and high tacrolimus groups was found. Thus, our studied was focused on these 6 genes with significant changes found in this verification study.

**Figure 4:**
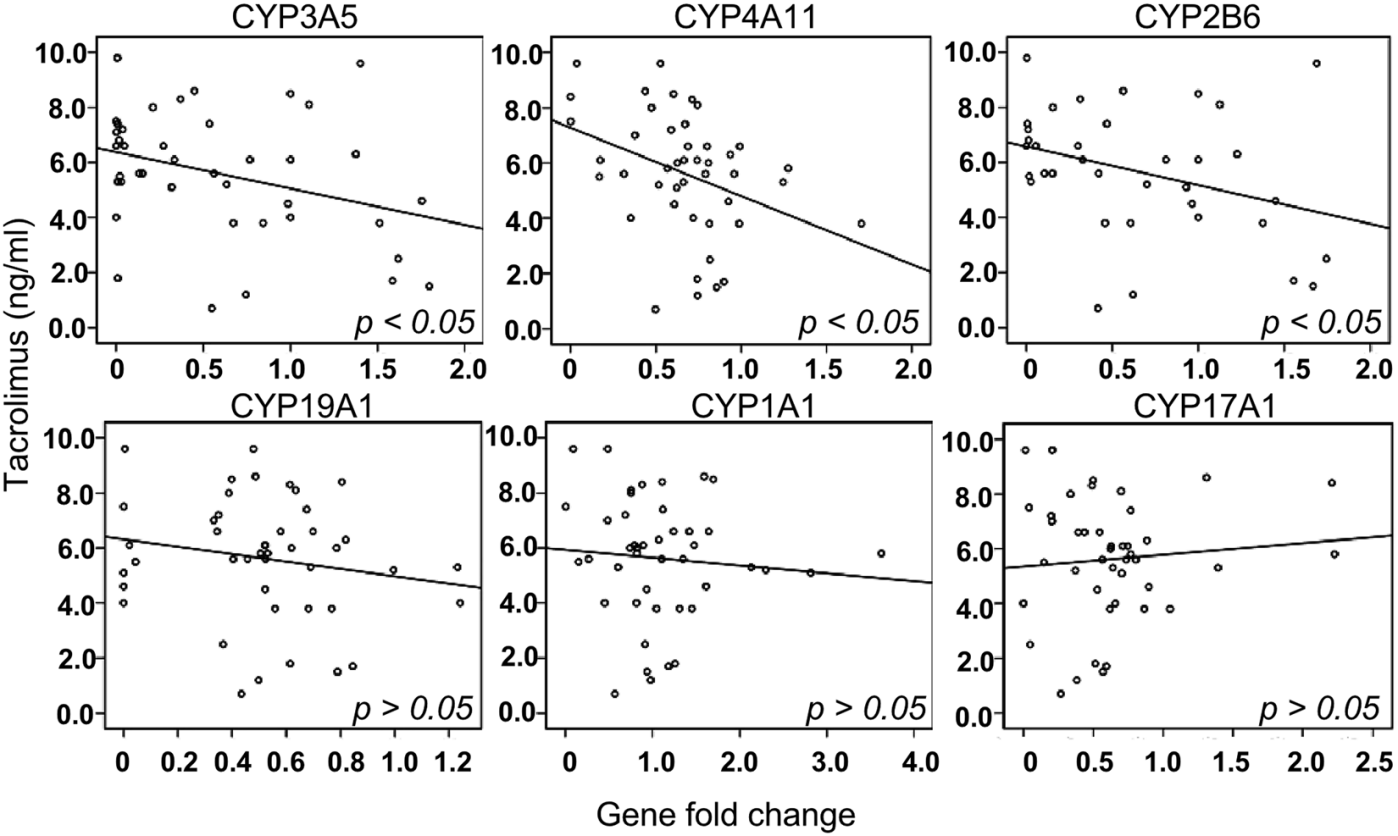
Linear regression analysis of the correlation between the blood tacrolimus level and the indicated gene expression level in liver transplant receipts.

We performed linear regression analysis with adjustment of age, sex, dose of tacrolimus and BMI to compare the relationship of the expression of these genes to the blood level of tacrolimus. The blood levels of tacrolimus were significantly inversely related to expression levels of *CYP3A5* (p < 0.05), *CYP4A11* (p < 0.05), and *CYP2B6* (p < 0.05) (Fig. 4 and Table 2). However, the blood level of tacrolimus did not have significant correlation with expression levels of *CYP1A11* (p > 0.05), *CYP19A1* (p > 0.05), and *CYP17A1* (p > 0.05), (Fig. 4 and Table 2). These results suggest that *CYP3A5*, *CYP4A11* and *CYP2B6* may have direct effects on metabolism of tacrolimus and *CYP1A11*, *CYP19A1*, and *CYP17A1* may contribute to other immunosuppressive drug metabolism. In addition, this finding indicates that other mechanisms may be involved in regulation the relationship between tacrolimus and expression of these genes.

**Table 2:** Linear regression analysis of the relationship between the blood level of tacrolimus and the gene expression level.

	Coefficients*		
	β	Standard deviation	*p value*
*CYP3A5*	−1.295	0.586	0.034
*CYP4A11*	−0.055	0.024	0.032
*CYP2B6*	−0.094	0.044	0.040
*CYP19A1*	−0.014	0.023	0.539
*CYP1A1*	0.187	0.259	0.476
*CYP17A1*	0.030	0.035	0.389

*Dependent variable: the blood level of tacrolimus, adjusted by age, sex, dosage of tacrolimus, and BMI.

### Enzyme assay for identifying subtracts of the identified enzymes

We next examined subtracts of the identified enzymes. Tacrolimus, mycophenolate mofetil, and methyl prednisolone in a mixture were incubated with recombinant human CYP17A1, CYP1A1, CYP4A11, CYP2B6, CYP3A5 and the enzyme activity was detected by detecting the levels of substrates using HPLC-MS analysis. As reported before^4, 5^, tacrolimus was a substrate of CYP3A5 (Table 3). We also found that mycophenolate mofetil was a substrate of CYP17A1 (Table 3). This finding revealed a previously unrecognized enzyme, CYP17A1, for mycophenolate mofetil metabolism.

**Table 3:** The enzyme assay results.

	Enzymes	Control group (R_T0_–R_T90_)/R_T0_	Experimental group (R_T0_–R_T90_)/R_T0_
**Tacrolimus**	**CYP3A5**	**−0.0920 ± 0.0828**	0.9866 ± 0.0068*
	CYP17A1	0.0017 ± 0.0582	0.0122 ± 0.0286
	CYP2B6	0.0410 ± 0.1047	0.0695 ± 0.0198
	CYP1A1	−0.0691 ± 0.0771	0.0056 ± 0.0674
	CYP4A11	−0.0753 ± 0.1091	−0.0791 ± 0.2259
**Mycophenolate mofetil**	**CYP17A1**	**0.0173 ± 0.0344**	0.2831 ± 0.0655*
	CYP2B6	0.0336 ± 0.0446	0.0238 ± 0.0196
	CYP1A1	0.3926 ± 0.0440	0.4955 ± 0.0425
	CYP4A11	0.5148 ± 0.1513	0.5343 ± 0.1589
**Methylprednisolone**	CYP17A1	−0.0242 ± 0.0564	0.0023 ± 0.0710
	CYP2B6	0.0015 ± 0.0426	−0.0031 ± 0.0361
	CYP1A1	−0.0105 ± 0.0030	0.0013 ± 0.0708
	CYP4A11	0.0102 ± 0.0760	0.0133 ± 0.1155

R_T0_: the relative peak area of substrate before reaction; R_T90_: the relative peak area of substrate at 90 min after reaction. *p < *0.05* compared to the control group. Data are analyzed from three independent experiments.

## Discussion

Liver cell damage could lead to liver cells entering peripheral blood^10^, which is supported by the evidence that CYP1A1, CYP2B6, CYP3A5, CYP4A11 could be detected in peripheral blood^15–18^. The significance of our study is to identify a correlation between expression levels of six genes, including the above 4 genes, and the blood level of tacrolimus and the liver function in liver transplant receipts. These results have clinical relevance for guiding application of immunosuppressive medications for solid organ transplant. However, since no published results reported the relationship between expression profiling of these metabolism-related genes identified in this study and the blood levels of immunosuppressive medications in any solid organ transplantation, more studies are needed to validate the efficacy of applying this gene expression profiling for selecting immunosuppressive medications and optimal dosage.

The enzyme assay showed that tacrolimus was a substrate of CYP3A5, as reported before^4, 5^. It has been reported that CYP3A5 genotypes affect the pharmacogenetics of tacrolimus^19, 20^ because different CYP3A5 genotypes exert different characteristics of the expression of CYP3A5^14, 21^. This evidence may contribute to the interindividual variability of the tacrolimus metabolism.

In addition to CYP3A5, our results showed that the blood level of tacrolimus was inversely related to the expression levels of CYP4A11 and CYP2B6. Furthermore, CYP1A1, CYP19A1, and CYP17A1 were significantly increased in the low tacrolimus group, as compared to those in the high tacrolimus group. It has reported that the level of serum creatinine was positively correlated with the level of 20-HETE, which indicates the role of creatinine in production of 20-HETE^22^. The level of 20-HETE is regulated by CYP4A11 and CYP1A1 because CYP1A1 and CYP4A11 are able to catalyze the arachidonic acid to 20-HETE^23, 24^. Thus, the level of 20-HETE may decrease as the level of tacrolimus becomes increased.

The CYP2B6 substrate selectivity comprises many diverse chemicals, and 7% drugs can be metabolized by CYP2B6^25^. It has been reported that expression of CYP2B6 is increased in primary human hepatocytes treated with tacrolimus^26^ and CYP2B6 may mediate metabolism of tacrolimus. However, tacrolimus could not be metabolized by the recombinant human CYP2B6 in the enzyme assay in this study. Tacrolimus is reported to be involved in the inhibition of the interferon gamma (IFN-γ) production and IFN-γ stimulates the expression of CYP2B6^27^. Thus, increased blood level of tacrolimus may lead to decreasing IFN-γ level and expression of CYP2B6. Therefore, expression of CYP2B6 may be related to liver injury.

CYP19A1 plays a role in metabolism of estrogen^28^ and is related to breast cancer development^29^. Expression of CYP19A1 is induced by TNF, IL-6 and IL-11^30^. Since tacrolimus suppresses IL-6^28^, the expression of CYP19A1 might be affected by tacrolimus.

It is known that a Phase II enzyme, UDP glucuronosyl transferases mediates mycophenolate mofetil metabolism^31^. A significant finding from this study is to identify CYP17A1, a Phase I enzyme, as a metabolic enzyme for metabolism of mycophenolate mofetil. CYP17A1 is a key enzyme of steroid metabolism by converting steroid to bile acid^32^, including hydroxylation of progesterone and pregnenolone and cholesterol metabolism^33^. Interestingly, steroid has been reported to play a role in inducing expression of CYP17A1, and abnormal expression of CYP17A1 is related to the level of low density lipoprotein cholesterol and high density lipoprotein cholesterol^34^. Furthermore serum lipid expression correlates with function and regeneration following liver transplantation^35^.

In this study, we assessed the correlation between expression levels of enzymes and the blood level of tacrolimus, but not with mycophenolate mofetil. Several clinical studies have shown the correlation between the dosage of mycopheno-lic acid and the acute rejection in kidney transplant patients^36, 37^. However, the correlation between the dosage of mycophenolic acid and toxicity is less convincing^38^. Furthermore, poor correlation between pre-dose mycophenolic acid concentration and dose-normalized mycophenolic acid area under the concentration-time curve is identified^39^. Based on this evidence, mycophenolate mofetil is typically administered as a fixed dose without routine monitoring of mycophenolic acid concentrations. However, it should be noted that pharmacokinetic between-subject variability of mycophenolic acid is an indication of requirement for therapeutic drug monitoring of mycophenolic acid. Thus, there are ongoing studies to investigate whether assessing inosine monophosphate dehydrogenase activity can be applied to predict mycophenolate mofetil dosage.

Since the majority of enzymes involved in drug metabolism are produced in the liver, it is important to evaluate the influence of gene expression profile in the graft liver and enzyme activity in liver transplant receipts. It has been reported that CYP3A4*1G genotype in the graft liver contributes to the frequency of acute cellular rejection after transplantation^40^. Based on this evidence, we speculate that the expression profile of genes involved in drug metabolism in the graft liver, including CYP genes and genes studied in our work, may have impact on pharmacokinetics of drugs in liver transplant receipts.

Peripheral blood can be easily acquired from patients and is widely used to evaluate clinical symptoms. The expression profiling of these drug metabolism-related genes was detected using RNA from the blood samples. Thus, the detecting method is convenient. The results from this study support the clinical application of the expression profiling of these drug metabolism-related genes for selection of immunosuppressive medications and the optimal dosages for organ transplant receipts.

## Methods

### Study population and immunosuppression protocol

Methods used in this study were carried out in accordance with the approved guideline by the Committees for Ethical Review of Research involving Human Subjects at Tianjin Medical University. Informed consent was obtained from all receipts.

A total of sixty-four consecutive liver transplant receipts who had undergone liver transplantation procedure from October 2013 to April 2014 in Tianjin First Center Hospital were enrolled in this retrospective study. All receipts are Chinese with the age range from 31 to 72 years (mean at 51 year-old) (Table 1). The exclusion criteria were incomplete immunosuppression regimen treatment.

The receipts were treated with a triple-drug immunosuppression regimen including tacrolimus, mycophenolate mofetil and methylprednisolone. Tacrolimus (Prograf, Astellas Pharma, Japan) was administered orally twice daily at an initial dose of 4 mg/day on the post operation day 2. Levels of tacrolimus in peripheral blood samples collected before the morning dose were examined everyday starting at the post operation day 2 using a microparticle enzyme immunoassay. The dosage of tacrolimus was individually adjusted based on receipts’ age, bodyweight, and the blood level of tacrolimus in previous day. The targeted blood concentration of tacrolimus is 7–10 ng/ml. The maximal tacrolimus dosage was 4 mg/day. The receipts received the same dose of mycophenolate mofetil and methylprednisolone. Mycophenolate mofetil was administered orally 500 mg before operation and 100 mg/day post operation. Methylprednisolone was administered as an intravenous dose of 1000 mg immediately after operation and 40 mg/day during post operation period. None of subjects took any medications that interfere with metabolism of these three immunosuppressive drugs.

Peripheral blood samples were collected before the morning dose administered at the third week after transplantation for examining trough levels of tacrolimus. The blood samples were stored at −80 °C until analysis. The clinical parameters were evaluated, including glutamic-pyruvic transaminase (ALT), glutamic oxalacetic transaminase (AST), alkaline phosphatase (ALP), glutamyltransferase (GGT), total bilirubin, serum creatinine, and blood urea nitrogen (Table 1).

### RNA extraction from the blood

RNA isolation from peripheral blood samples was performed using Trizol (Invitrogen, Paisley, UK) reagents according to the manufacturer’s instruction. The concentration and purity of RNA were evaluated by NanoDrop 2000 UV-Vis Spectrophotometer (Thermo Scientific). Complementary DNA (cDNA) was synthesized by reverse transcription. Total RNA (200 ng) was reverse-transcribed into cDNA by using the RNA to cDNA premix. Each sample was reverse-transcribed using the RT^2^ First Strand kit (Qiagen).

### Human Drug metabolism RT^2^ Profiler PCR Array

These receipts were divided into low (2–5.9 ng/ml) and high (6–15 ng/ml) tacrolimus groups. The gene expression levels were examined in four receipts from the low tacrolimus group with the blood tacrolimus levels of 2.7, 3.9, 4.1 and 4.2 ng/ml and four receipts from the high tacrolimus group with the blood tacrolimus levels of 6.0, 6.3, 9.3 and 11.3 ng/ml, using Drug Metabolism RT²Profile PCR Arrays.

Human Drug metabolism RT^2^ Profiler PCR Array (Qiagen) contains 84 genes involved in metabolism of drugs, including Phase I Enzymes and Phase II Enzymes in absorption, distribution, metabolism and excretion of drugs, 5 housekeeping genes (HKGs; ACTB, B2M, GAPDH, HPRT1 and RPLP0), a genomic DNA control (GDC), 3 reverse-transcription controls (RTC), 3 positive PCR controls (PPC). This array was performed through three sub-arrays, PAHS-002ZE, PAHS-068ZE, and PAHS-069ZE (Qiagen)

cDNA of from each sample was mixed with RT^2^ qPCR Master Mix containing SYBR Green (Qiagen) and aliquoted into each well of the custom-made array. Gene analysis was performed using the ABI 7900 instrument (Applied Biosystems, Foster City, CA), according to the manufacturer’s instructions. Gene expression levels were normalized by the housekeeping genes by using the following equation:

$${\rm{Fold}}\,{\rm{change}}={2}^{-{\rm{\Delta }}({\rm{\Delta }}\mathrm{Ct})}.$$

where ΔCt = Ct (target gene) − Ct (housekeeping genes), and Δ(ΔCt) = ΔCt (low tacrolimus group) − ΔCt (high tacrolimus group). The relative expression levels of these eighty-four genes were compared between the low and high tacrolimus groups.

### Validation by real time-PCR

We used custom-made RT² Profiler™ PCR Array to validate the result from human drug metabolisms RT² Profiler™ PCR Array using an ABI 7900 instrument. Gene expression levels with higher than 3-fold change (Supplemental Table 1) were verified by real-time PCR analysis using blood samples from sixty-four receipts.

### Enzyme assay

Recombinant human CYP1A1 and CYP4A11 (Corning) and CYP2B6, CYP17A1 and CYP3A5 (Cypex) were separately incubated with TAC (50 ng/ml), MMF (1 μg/ml), and MP (12 μg/ml) in 500 μl of PBS buffer, pH 7.4, containing 1 mM NADPH, for 90 min at 37 °C. The reaction was terminated by adding an 3 volume of ice cold acetonitrile. Carbamazepine was used as an internal standard. The mixture was centrifuged at 10,000 g for 10 min at 4 °C. The supernatant was collected and analyzed by HPLC-MS (Agilent America. MS, API4000, America). The peak area of 0 min and 90 min represents the levels of the content of substrate. Incubations were performed in triplicate and negative controls were run in parallel.

### Data analysis

Statistical analysis was performed using Statistic Package for Social Science (SPSS) 19. Independent sample t-test was applied to compare data from two groups. Relationship between the blood levels of tacrolimus and the expression levels of genes were examined using linear regression analysis that was adjusted by age, sex, dose of tacrolimus, and BMI. The level of statistical significance was set at *P* < 0.05. Data were presented as mean ± standard error of the mean.

## Electronic supplementary material

Supplemental table 1
