# Supplementary material for: The correlation between the expression of genes involved in drug metabolism and the blood level of tacrolimus in liver transplant receipts
Source: Sci Rep. 2017 Jun 13;7:3429. doi: 10.1038/s41598-017-02698-w (PMC5469744; doi:10.1038/s41598-017-02698-w)
Supplement: Supplementary file 1 — Supplemental table 1 [file 41598_2017_2698_MOESM1_ESM.pdf]

The correlation between the expression of genes involved in drug metabolism and the blood level of tacrolimus in liver transplant receipts

Jianhai Wang[1], Keqiu Li[1], Xiaoning Zhang[1], Dahong Teng[2], Mingyan Ju[1], Yaqing Jing[1], Yuxia Zhao[1], Guang Li[1*]

[1] Basic Medical College, Tianjin Medical University, Tianjin, 300070, China
[2] Department of Hepatobiliary and Liver transplantation Surgery, Tianjin First Central Hospital, Tianjin, 300192, China

Running title: Gene expression and the blood level of tacrolimus in liver transplant receipts

[*]**Corresponding author:**

Guang Li
Professor
Basic Medical College
Tianjin Medical University
22 Qixiangtai Road, Heping District
Tianjin, 300070, China
Email: lig@tmu.edu.cn

Phone: 011-86-22-83336839

Keywords: drug metabolism; gene expression; liver transplant; mycophenolate mofetil; organ rejection; tacrolimus.

Supplemental Table 1. Genes with significant changes of the expression levels identified in Drug Metabolism RT²Profile PCR Arrays

	Gene	Fold Change*	*p value*
Phase I enzymes	*CYP8B1*	3.7524	0.040004
	CYP4F3	3.4386	0.009126
	CYP4A11	4.0740	0.034044
	CYP3A5	3.8184	0.049760
	CYP3A43	3.6358	0.025309
	CYP2C9	15.4349	0.026992
	CYP2C19	5.5827	0.015599
	CYP2B6	4.2845	0.048768
	CYP2A13	3.9677	0.032291
	CYP1A1	3.6028	0.042150
	CYP19A1	5.9401	0.018675
	CYP17A1	22.2836	0.045420
	ALDH7A1	3.6878	0.026845
	ALDH5A1	3.6389	0.022865
	ALDH1A3	3.1633	0.028930
	ADH7	4.8964	0.045980
	ADH6	8.2639	0.037700
	ADH5	3.9610	0.043810
	ADH1C	7.5629	0.007500
	ADH1B	4.6237	0.026390
	ADH1A	7.2141	0.026640
	PTGS2	4.7029	0.021510
	XDH	3.9166	0.047120
	PTGS1	4.2884	0.009490
Phase II enzymes	*GAD1*	3.8351	0.044790
	SULT1B1	4.7004	0.043540

*Expression levels in the low tacromulis group are compared to those in the high tacrolimus group.
